# Supplementary material for: EEG dynamical network analysis method reveals the neural signature of visual-motor coordination
Source: PLoS One. 2020 May 27;15(5):e0231767. doi: 10.1371/journal.pone.0231767 (PMC7252646; doi:10.1371/journal.pone.0231767)
Supplement: S1 File — In the Supplementary Information, we performed systematic analysis for a range of threshold to define functional connectivities in the alpha and gamma band. (PDF) [file pone.0231767.s001.pdf]

Supplementary Information
Parameter sweep of threshold

Xinzhe Li, Bruno Mota, Toshiyuki Kondo,
Slawomir Nasuto, Yoshikatsu Hayashi

In our study, we applied a single fixed threshold which is 4×10^{-4} on the Phase-Locking Value (PLV) to define functional connectivity networks for a given EEG data set. This supplementary material describes how we calculated the distributions of PLVs, and extracted the graphical and dynamical properties of functional connectivity networks with different thresholds in order to compare with the results in Fig 9 and Fig 14 and provide a more comprehensive picture of the functional connectivity networks which would depend on the threshold employed.

The PLVs of the brain signals follow a log-normal distribution, whose logarithm follows an Gaussian distribution as shown in S1 Fig 1. The log-normal distribution can be explained by the definition of the PLV, which measures the differences of the phases based on complex exponential function.

The threshold parameters were determined by the log-PLV distribution. We estimated the key percentiles of the log-PLV distributions by estimating the percentiles of the representative distributions whose mean and standard deviation are the closet to the trial-wise averages. Large differences of the percentiles were found between different frequency bands. In alpha band, 10% percentile is approximately 4.3×10^{-4}, while in gamma band, it is the percentile of 0.7%. Some key percentiles are summarized in S1 Table 1. For convenience, the value of percentiles and thresholds are in unit of 1×10^{-3} in the following texts.

	0.7%	5%	10%
Alpha band	0.10	0.28	0.43
Gamma band	0.43	1.10	1.61

S1 Table 1: The approximate percentiles of the log-PLV distribution (in unit of 1×10^{-3}). Distributions of different trials show variable values of mean and standard deviation. Therefore we estimated these percentiles by looking for the representative distribution whose parameters are the closet to the trial-wise averaged parameters, and then estimating the percentiles from that distribution. These percentiles show very large differences between the alpha and gamma band. These estimations are coarse-grained but they are informative for the determination of the parameter range in the threshold sweep.

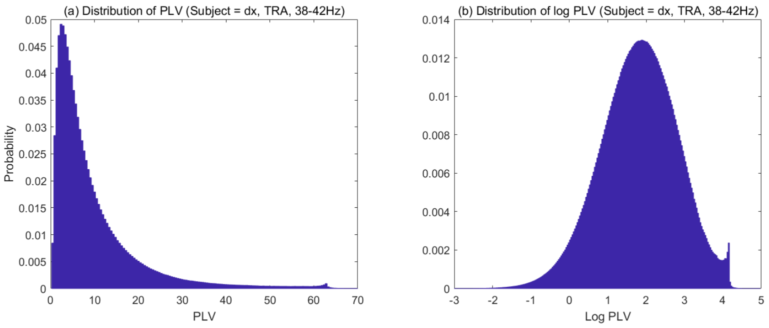

S1 Fig 1: (a) The PLV distribution of the Tra trials of one participant in gamma band. (b) The distribution of log PLVs of the same trial set. The PLVs of all the trials and participants subject to the log-normal distribution, which can be explained by the measurement method of PLVs. The kurtosis of the log distribution is related to the frequency band, the alpha band has the largest kurtosis (3.32, average of all trials and participants), and the gamma band has the smallest kurtosis (3.00, average of all trials and participants). There is no significant difference of kurtosis between different experimental conditions.

According to those percentiles, we set 4 values of threshold for each frequency band. In alpha band, we set 0.1, 0.2, 0.3 and 0.5 as threshold. In gamma band, we set 0.7, 1.0, 1.3 and 1.6 as threshold. These threshold values cover the range approximately from 10% to 1% of the log-PLV distributions in both frequency bands.

S1 Fig 3-6 show the graphical properties and the dynamical properties of the functional connectivity network with different threshold. The distribution of the prime eigenvector inner product follows the bimodal distribution, therefore we perform the same measurement as in the Results section of the article, which is the frequency of prime eigenvector < 0.01 (the 0 event) and > 0.99 (the 1 event). The 0 and 1 events correspond to the two peaks of the bimodal distribution. Notably, in the case of gamma band with the threshold of 1.3 and 1.6, the distribution of the inner product of prime eigenvector also follows the bimodal distribution but the right peak is in the interval of $[0.98, 0.99)$ instead of the $[0.99, \infty)$, as shown in S1 Fig 2. Therefore, the 1 event is defined on the peak interval which is $[0.98, 0.99)$ for these two thresholds in gamma band. As a comparison, we also showed the frequency of event within $[0.99, \infty)$ in S1 Fig 7.

All the results suggest that both graphical properties and dynamical measurements keep the significance of differences between conditions regardless of the change of the threshold, comparing to Fig 9 and 14 in the article. However, the change of the threshold does have an interesting effect on the frequency of inner product event. In S1 Fig 4, frequency of 1 of VO is significantly smaller than the other two conditions when threshold is 0.1.

However, when the threshold increased to 0.3 and 0.5, VO is significantly

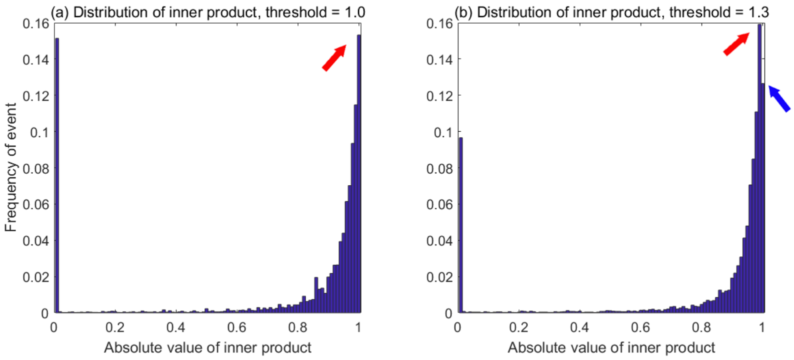

S1 Fig 2: Distribution of eigenvector inner product of the same trial in the gamma band with different thresholds, which are marked in figures (in unit of 1×10^{-3}). Both distribution follow bimodal distribution, but their right peaks fall on different intervals, as shown by red arrows. The blue arrow in the subfigure (b) indicates the interval $[0.99, \infty)$, which is not the peak anymore when threshold increases from 1.0 to 1.3 (in unit of 1×10^{-3}).

larger than the other two conditions. The same phenomenon is also be observed in gamma band. S1 Fig 6 shows that frequency of 1 of MO is significantly lower than the other two conditions when threshold is 0.7 and 1.0. At a higher level of threshold of 1.3 and 1.6, frequency of 1 of MO became significantly larger than the others. What's more comparing the frequency of 1 of gamma band in Fig 14 and S1 Fig 6, it can be found that the MO is significantly higher than the other two conditions when threshold is 0.4 in Fig 14, while MO became lower than the others as the threshold increased in S1 Fig 6. This interesting phenomenon indicates that the network has different layers of active connections and clusters, and they emerge one by one as we tuning the threshold as a filter of connection strength. In future work we will further study this phenomenon.

In conclusion, the significance of differences of the graphical properties and the prime eigenvector inner product measurement between conditions as found in our results (Fig 9 and Fig 14) were not affected by the threshold changing within the 10% percentile of the log-PLV distribution.

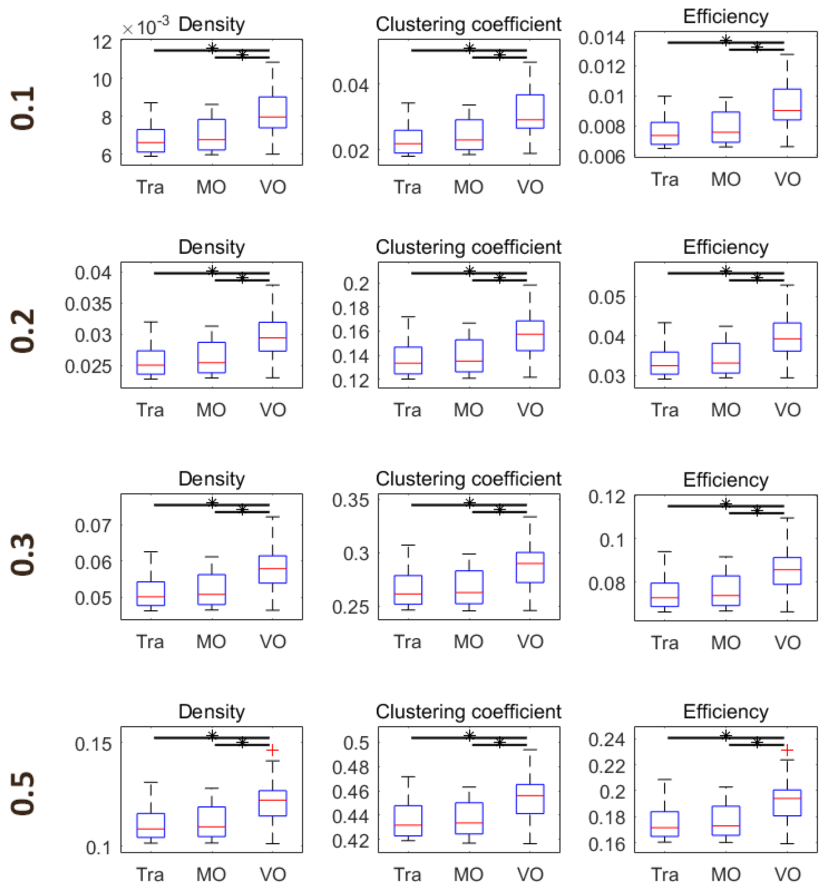

S1 Fig 3: The graphical property of the functional connectivity networks in alpha band with different thresholds. The values which are more than 1.5 times the interquartile range from the box are marked with red + sign. Each row refers to one threshold parameter, which is shown on the left side (in unit of 1×10^{-3}). The left column shows the network density, the middle column shows the clustering coefficient, and the right column shows the network efficiency, which is the reciprocal of the shortest path length. The horizontal bars with stars indicate pairs showing significant difference (paired Wilcoxon signed rank test, $\alpha = 0.05$).

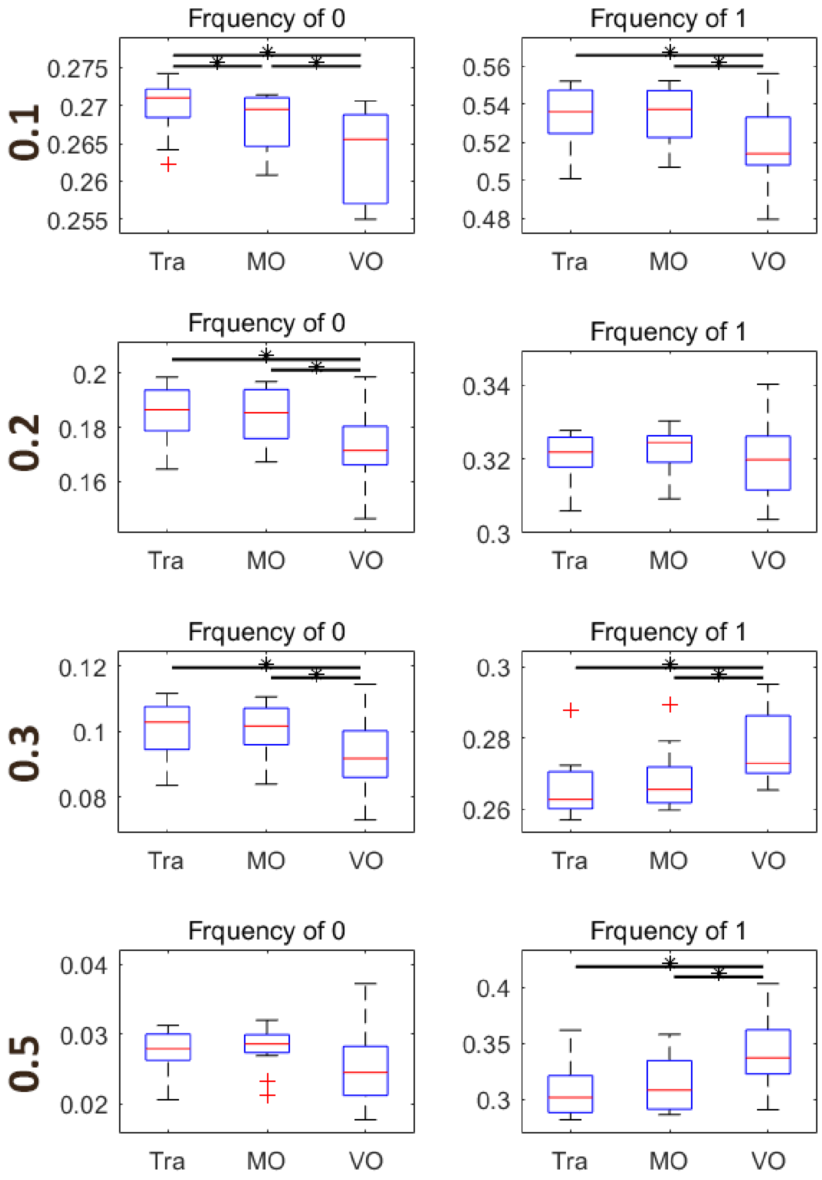

S1 Fig 4: Comparison of the probability of inner product of prime eigenvectors in alpha band. The values which are more than 1.5 times the interquartile range from the box are marked with red + sign. Each row refers to one threshold parameter, which is shown on the left side (in unit of 1×10^{-3}).Event 0 is defined as inner product within $(-\infty, 0.01)$, event 1 is defined as inner product within $[0.99, \infty)$. The horizontal bars with stars indicate pairs showing significant difference (paired Wilcoxon signed rank test,$\alpha = 0.05$).

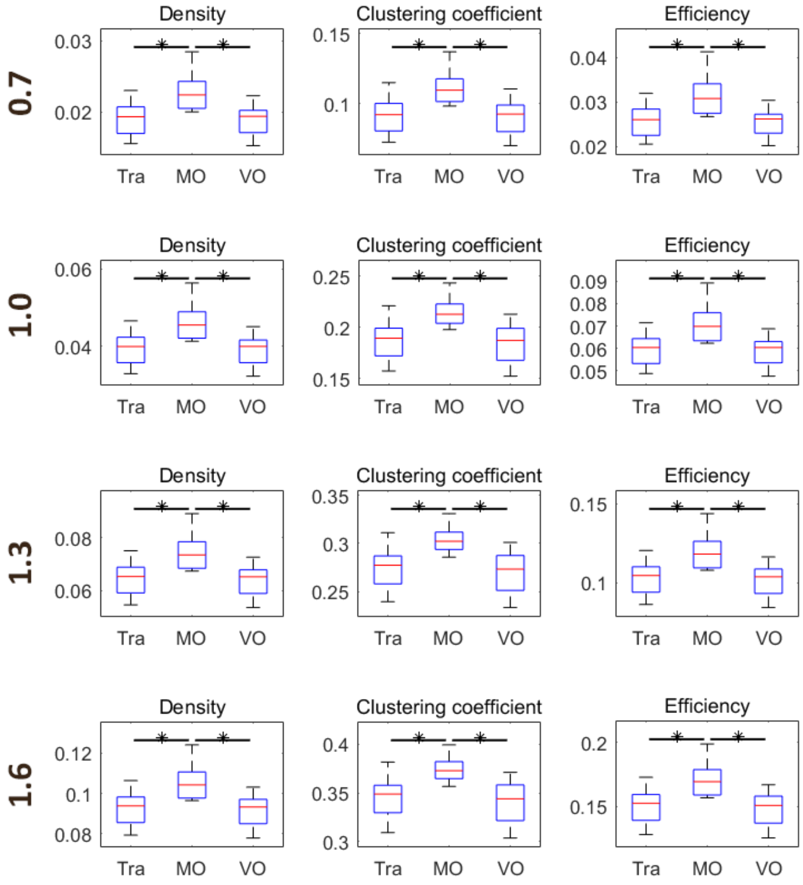

S1 Fig 5: The graphical property of the functional connectivity networks in gamma band with different thresholds. Each row refers to one threshold parameter, which is shown on the left side (in unit of 1×10^{-3}). The horizontal bars with stars indicate pairs showing significant difference (paired Wilcoxon signed rank test,$\alpha = 0.05$).

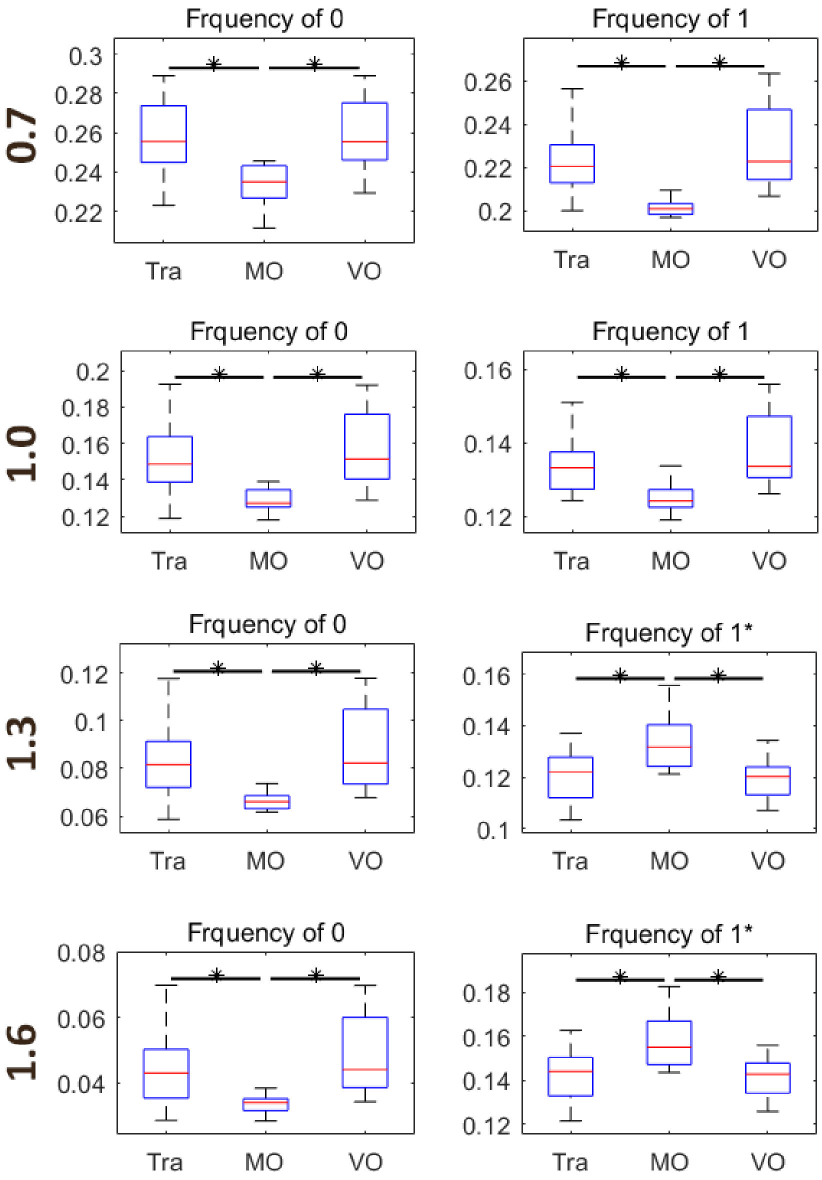

S1 Fig 6: Comparison of the probability of inner product of prime eigenvectors in gamma band. Each row refers to one threshold parameter, which is shown on the left side (in unit of 1×10^{-3}).Event 0 is defined as inner product within $(-\infty, 0.01)$. Due to the differences in distributions as discussed previously, event 1 is defined as inner product within $[0.99, \infty)$ in the case of threshold being 0.7 and 1.0, and defined within $[0.98, 0.99)$ in the case of threshold being 1.3 and 1.6 (marked by star). The horizontal bars with stars indicate pairs showing significant difference (paired Wilcoxon signed rank test,$\alpha = 0.05$).

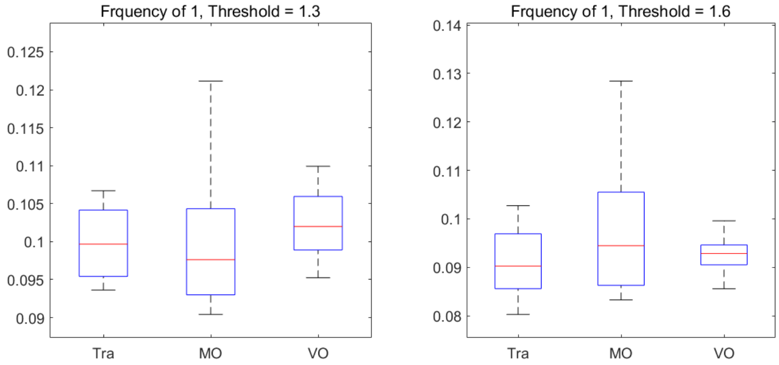

S1 Fig 7: The frequency of event 1 which defined as inner product within $[0.99, \infty)$ in gamma band with thershold 1.3 and 1.6. There is no significant difference between them.
