# Supplementary material for: EEG dynamical network analysis method reveals the neural signature of visual-motor coordination
Source: PLoS One. 2020 May 27;15(5):e0231767. doi: 10.1371/journal.pone.0231767 (PMC7252646; doi:10.1371/journal.pone.0231767)
Supplement: Attachment — Submitted filename: Response to Reviewers.docx [file pone.0231767.s002.docx]

Dear Editor and Reviewers,

We would like to thank you for all the insightful comments, which helped us improve the manuscript and the presented research. According to the comments from the reviewers, we have revised the manuscript as follows. The reviewers’ comments are in bold and our responses are in blue. The corresponding changes of the manuscript are in blue italics.

1. Reviewer #1 wrote **"The article also presents the results of the standard topological analysis along with the newly proposed change detection method on the visual-motor EEG data. This analysis may well be worthy of publication, but the emphasis of the paper needs to be considered carefully. Is this a paper about analysis of the visual-motor EEG data using quite standard methods or is it a paper about a new method for the analysis of the dynamic evolution of neural network? If it is the former, then the emphasis of the paper should be on that. If it is the latter, then the advantages of the proposed method over the competing ones need to be explained better."**

In this study, both eigenvector-based dynamical analysis method and the analysis results of EEG experiment are our original contributions. We agree that we did not emphasise sufficiently the analysis results of EEG functional connectivity. Therefore we revised Abstract, Introduction and other parts of the manuscript to emphasise our contribution of the EEG functional connectivity study. We revised the Abstract as follows:

*Human visual-motor coordination is an essential function of movement control, which requires the interaction of multiple brain regions. Understanding the visual-motor coordination is important for improving the therapy of motor disability. However, its underlying transient neural dynamics is still largely unknown. In this study, we applied an eigenvector-based dynamical network analysis method to investigate the functional connectivity calculated from electroencephalography (EEG) signals under visual-motor coordination task and to identify its meta-stable states dynamics. We first tested the method on a simulated network to evaluate it in comparison with other dynamical methods, which demonstrated that the eigenvector-based dynamical network analysis method was able to correctly extract the dynamical feature of the evolving simulated networks. Subsequently, the eigenvector-based method was applied to EEG data collected under a visual-motor task experiment. In the EEG study, the results of both topological analysis and the eigenvector-based dynamical analysis were able to distinguish different experimental conditions of visual tracking task. With the dynamical analysis, we showed that different visual-motor coordination states of human can be distinguished by investigating the meta-stable states dynamics of EEG functional connectivity.*

2. Reviewer #1 wrote **“Looking at the results, the simulation shows that indeed the proposed method can pinpoint the time of dynamic changes in the connectivity of neural networks. However, the evidence for the proposed method showing an advantage over the dynamical clustering methods is not convincing. In fact, looking at the results presented in Fig.3-7, the competing methods seem to provide more information, i.e., not only a time point of change in the main cluster but also the number of clusters and their evolution over time. Both the proposed method and the ones compared demonstrate some minor issues. Disappointingly, it also seems that the proposed method is limited to finding the point in time when the dynamic change in the connectivity of neural network takes place, with the standard methods being applied for topological analysis of the networks as shown in Fig.9. Thus, I cannot agree that the proposed method, as it is explained and presented in the paper, is worthy of publication. Perhaps, if there were other advantages to the proposed method and they are highlighted clearly, it would be a different matter.”**

The eigenvector-based method in this paper was proposed to detect the meta-stable states transitions of the EEG functional connectivity network. The simulation performed in the beginning has two purposes. First, the simulation results demonstrate that the eigenvector-based method works well when detects the changes of the cluster structure in the network. Second, the comparison between the eigenvector-based method and the standard clustering methods demonstrates that the eigenvector representation of the prime eigenvector is easy to be compared between different instants of time. Although the two standard clustering methods provide more information of the small clusters in the network, the complex information of the clusters also make them not suitable for anaylsing the long time series of network, as we discussed (Line 484-502):

*In a complex network where several structural changing events are taking place, it would be hard to track the network dynamics with those clustering algorithms. By comparing the two prime eigenvectors from different moments, however, the changes of the largest cluster can be recovered in detail without losing continuity, as the prime eigenvector always correspond to the largest cluster in the network. In addition, the simple eigenvector representation of the prime cluster enables easy and direct comparison between the networks at different time instants by performing the inner product. For the hierarchical and modularity clustering, however, the complicated clustering information prevents the direct temporary comparison, which makes them not suitable for the analysis of long time series. The simplified representation of neural signal features is not uncommon. One example is the event-related desynchronization (ERD), which is known as the decreasing of the EEG power spectrum intensity around 10Hz, and has been reported to be the neural signature of attention and motor intention [34, 37, 38]. Even though Fourier spectrum itself over frequency range can provide a wide range of cognitive498information, ERD, which is a single scaling parameter, has been used as an indicative parameter. Our eigenvector-based method is designed to provide a single parameter in the temporary domain which can indicate the most prominent feature of the dynamical changes in cluster structure of the network.*

In addition, the simple eigenvector representation also facilitates the online application of the EEG signal in the BCI. Please refer to response 5.

3. Reviewer #1 wrote **“There are some other issues with the paper which are listed below. The abstract is too general and needs to be more specific about what kind of analysis is provided by the new method, i.e., what its output is. Reference [32] is very general describing many dynamical clustering methods. Explain why these particular methods were chosen for comparison. Substitute [32] for another reference if necessary. In Section "Eigenvector based dynamical analysis" explain how the values of different constants were chosen. In Fig.9, indicate where (a) and (b) are and increase font size in Fig.9-10. Add a reference for standard topological analysis in Section "Topological analysis". There are some minor typos in the article, which need to be corrected.”**

We have revised the abstract to better clarify and specify the focus and the method of this research please see response 1. In line 175 we have stated that we are comparing the eigenvector method with the hierarchical clustering and modularity clustering methods. The Reference [32], which has been now labelled as [28], has a detailed introduction on the hierarchical clustering method. We moved Reference [29] which is Newmann's work of modularity clustering to the same position as a reference for the modularity method we applied here. We quote the text here (Line 174-177):

*Apart from the proposed eigenvector method, two dynamical clustering methods, hierarchy clustering and modularity clustering, were also applied on the simulation data for comparison [28, 29]. The same time series of adjacency matrix which were based on the phase difference were fed into these two clustering methods.*

4. Reviewer #2 wrote **"Fundamentally, the experimental paradigm used is an ill-suited application of the proposed method. The method is presented as a technique to detect dynamical transitions in network synchronisation, but *there are no transitions in the experimental paradigm*. Subjects are instructed to track a target in continuous circular motion -- what kind of transition do we expect to see here? The choice of experimental paradigm is odd and, if the authors had any particular hypothesis for investigating synchronisation transitions in this setting, it should be clearly stated. The paper would make a much stronger case if the key results related to the proposed method (that there are some differences in transition frequencies between TRA, MO, and VO) were more adequately interpreted and put into context. While the method is interesting and potentially useful, I fail to see how it reveals any neural signature of visuomotor coordination in the context of this particular experimental paradigm. Describing precisely what the underlying hypothesis about brain function was, and how the method sheds light on that hypothesis, would make the paper much more accessible to the neuroscience community. "**

The dynamical analysis described in this paper was developed to explore the dynamical features of the EEG functional connectivity network in the visual-motor coordination. Our eigenvector-based method showed the intrinsic transitions between meta-stable states during the network evolution rather than the artificial experimental-embedded transition such as the change of the behavioural experimental conditions. There have been many studies (including Cabral’s work, which would be discussed in the next response) in the meta-stable state dynamics of brain signal as we cited Lehmann's work in the Introduction section (Line 45-49):

*Lehmann et.al. have introduced the EEG microstates, which utilize the global field power (GFP). The EEG microstates measure the amplitude of EEG signals over the scalp, and characterizes the unique topological patterns of the GFP into different ``states''. EEG microstates focus on the alpha band, and it requires a fitting of the GFP topographies into pre-determined patterns.*

In this work, we would like to show that such meta-stable states transition dynamics also happen in the visual-motor coordination process and the dynamics of these meta-stable states does relate to different visual-motor coordination states.

5. Reviewer #2 wrote "**Using eigenvectors to find structure in networks in a well-known field of research. More than 10 years ago, it was already explored in the work of Newman[1] and many other network scientists. More recently, and more directly relevant to this paper, this technique was applied by Cabral and colleagues [2] to the analysis of dynamical connectivity of fMRI data. Cabral's method is virtually identical to the one presented here: compute the phase of the Hilbert transform of each channel, compute some form of functional connectivity (phase coherence in Cabral's work, pseudo-PLV here), and take the inner product between leading eigenvectors of these time-varying connectivity matrices.**

**The authors need to make clear what the novel elements of their method are, and cite the relevant literature. If there are any new elements in the proposed method, they should be clearly stated, and the benefits of introducing them should be assessed. If there aren't, the authors should spend less space explaining the method and simply refer to the literature.**"

Thank you very much for your comment. We were not aware of Cabral's work, which applied similar eigenvector technique for dynamical functional connectivity analysis. However, there are several differences between their method and ours. Discussion of those differences was added in the following manner (Line 728-754, Fig 15):

*The idea of studying the network evolution with the prime eigenvector has been applied before. Cabral et. al. [47] discovered the states transitions in the rest functional connectivity of fMRI signal by similar prime eigenvector representation of the network, and showed that the state transitions are related to the cognitive performance of older adults. However, to our knowledge, this is the first study that applied the prime eigenvector method on the EEG dataset, and demonstrates the relation between the meta-stable states of EEG functional connectivity and the visual-motion coordination of human. There are also a number of differences between their techniques and ours. In the paper of Cabral et. al., they used cosine of phase difference as the phase-locking measurement, which can only detect in-phase and anti-phase synchrony. In our study, we applied Euclidean distance of angular speed vector to measure the phase synchrony by which any constant phase lag would be recognised as phase-locking. As we discussed in the Method section* (Please see the next response below)*, the angular speed difference could be more sensitive than the phase coherence in some cases, and the property of the Euclidean distance is able to prevent certain type of miss-recognition of synchrony event. Furthermore, the inner product time series analysis used in this study could be potentially applied on the online processing of brain-computer interface (BCI) [48,49] as a monitoring method to detect meta-stable states. BCI is a system, for example, which can capture brain signals to assist subjects who have movement or communication disability, which therefore requires an online brain signal analysis method. While Cabral’s work studied each and every eigenvector of network snapshot through the whole time scale, by focusing on the transitions on the diagonal our technique can be easily adopted as an online processing method. In Fig 15 we showed the accumulated event frequency time series of the 3 experimental conditions, which is an example demonstrating the online-adopted analysis. This figure shows that the three conditions follows the same quantitative relations as the statistical results shown in Fig 14, which could be used to identify different visual-motor states of subject.*

6. Reviewer #2 wrote "**The quantity in Eq. (4) is not an actual phase-locking value. The PLV is related to the average difference between the phases (as correctly described in Eq. (1)), while Eq. (4) i) takes the difference between complex exponentials instead of raw phases, and ii) takes the square of these differences for each timestep. Both of these could dramatically change the behaviour of the measure. Why not simply take the actual PLV, using the standard formula?**

**PLV(m,n) = (1/T) | \sum_{t=1}^T e^{i (\phi_{m,t} - \phi_{n,t}) } |**

**This would make the paper's results much easier to interpret, and it would be much less confusing for readers. The dynamical connectivity matrix can be defined using the same sliding window method that is currently used, and the method should be just as applicable.**"

We have clarified why we applied the Euclidean distance of angular speed vector as the phase synchrony measurement. Please not this response also responds for the previous comment. (Line 100-114) :

*This PLV measurement is similar to another widely-used technique called phase coherence, which is defined as PLV_{m,n} = \frac{1}{T} | \sum_{t=1}^T e^{i (\phi_{m,t} - \phi_{n,t}) } |. However, there are several differences between the phase coherence and the PLV based on the Euclidean distance used in this study. First, the main difference is that phase coherence measures the mean phase difference while our method measures the angular speed difference, which could be more sensitive to synchronization changes than the former in some cases. The other difference is that the projections with the opposite directions of the complex phase difference vector would be offset by each other, and those with the same directions would be enhanced during the calculation of phase coherence, while the Euclidean distance always add the angular speed differences up. For example, an intersection of two phase time series could be incorrectly identified as phase-locked, because the imaginary parts of complex phase difference vector before and after the intersection point would be offset by each other while the real parts would be added up, therefore the final PLV would be a relatively high value. However, the Euclidean distance is not normalized, which makes it always require a threshold as the benchmark.*

7. Reviewer #2 wrote "**In line 281, the authors say that the matrix was binarised 'with a visual-based threshold.' I can't emphasise enough how important the consequences of this threshold choice are. There is also an extensive literature on the impact of such choices, see for example Ref. [4] among many others. If thresholding is absolutely necessary, then authors should conduct extensive parameter sweeps and controls to ensure that the results are genuine and not an artefact of the 'visual-based threshold.'** "

We have clarified how we selected the threshold by visual-inspection. The motivation of applying the threshold is to focus on certain type of synchrony relation that we are interested in. The resulting threshold is a fixed value and the same for all the conditions, which should not generate threshold-related artefact in the result. In the following text we described the synchrony relation we investigated in this study (Line 115-129, Fig 1) :

*The threshold used in this study was determined through visual inspection. We reviewed the raw time series of the angular speed, and calculated the phase locking value for the pair-wise signals which were found to be phase-locked in the raw time series. Figure 1 shows a segment of angular speed time series from the actual EEG data, which further demonstrate the selection of the threshold. The two arrow pairs in the figure indicate two example segments of phase-locking. The segments of channel F1 (blue triangles in the figure) and FZ (yellow triangles in the figure) between the magenta arrow pairs are very close to each other during this certain period of time, so they are identified as phase-locked during this period of time. The segments of channel P1 (purple hexagon) and PZ (green hexagon) between red arrows provide another example of the phase-locking channel pairs. With a large number of samples having been reviewed and calculated, the proper threshold (4 x 10^-4) for the binary functional connectivity matrix was determined. At this stage, a time series of binarised adjacency matrix was obtained, which represents the evolving functional connectivity of brain signal.*

8. Reviewer #2 wrote "**The results of the topological analysis in Fig. 8 do not seem properly controlled. Naturally, a denser network will have both a higher clustering coefficient and shorter path lengths -- not necessarily because of fundamentally different properties of the network-generating process, but *simply because the network is denser*. To make the findings interpretable and unambiguous, the results for clustering coefficient and efficiency should be compared against suitable null models (for example, random Erdos-Renyi networks of the same density). Also, there is no mention whatsoever of corrections for multiple comparisons, which need to be applied when conducting an analysis on several measures in several frequency bands.**"

In response to the question that whether the two topological properties, network efficiency and clustering coefficient, are just function of the network density, we used random networks to generate null model with the same network density, and showed that the EEG functional connectivity has a different topological property distributions from the random network. (Line 533-547, Fig 10)

*In order to test whether the clustering coefficient and the network efficiency is only related to the network density, we compared these two properties between the functional connectivity networks calculated from the EEG signals and random networks. We generated random networks whose have the same network density as the EEG functional connectivity. Fig 10 shows topological property distributions of the functional connectivity of tracking trials in alpha band and the random networks with the same density. It can be found from the figure that distributions of both network efficiency and clustering coefficient are different between the functional connectivity and the random network. The fact that connections are randomly placed in the random network explains that the mean clustering coefficient of random network is smaller than the EEG functional connectivity. It also suggests that there are more isolated nodes in the EEG functional connectivity, which leads to lower efficiency in the random network as shown in Fig 10 (a). This result shows that although the clustering coefficient and the network efficiency are strongly related with the network density, the EEG functional connectivity network has its own topological structure different from the random network.*

9. Reviewer #2 wrote **"The study claims to study synchronisation in 8 Hz oscillations using a window of 40ms. *That is less than a third of a single period* of the oscillation, so phase-locking values are likely to have a very strong bias. This is also an issue (although less dramatic) for the other frequency bands. The authors should take this bias into account for their analyses, as well as investigate (and report) the effect of different window sizes."**

In our analysis, we quantify the phase-locking values between the oscillators by measuring the angular speed differences between them. The angular speeds are not expected to be affected by the period, therefore the time window size should not add bias into the phase-locking value measures.

10. Reviewer #2 wrote **"In the discussion of analysis results in line 564, what does it mean for 'the frequencies of 0 event and 1 event' to be 'highly consistent with the topological properties'? The topological properties make no statement about network dynamics, and no null model or hypothesis is presented to compare those two."**

We are sorry for this confusion. Here we want to point out that the TRA and VO in alpha band are significantly different in both topological properties and the meta-stable states transition frequencies, and the same for the TRA-MO in gamma band. We have revised the text to make the expression less confusing (Line 599-602):

*Comparing with the topological properties shown in Fig 9, it can be seen that Tra-VO pair in the alpha (8-12Hz) and Tra-MO pair in the gamma (38-42) are significantly different in terms of both topological and dynamical properties.*

11. Reviewer #2 wrote **“The details of the simulations could be more clearly specified. For example the coefficients a_{m,n} or the initial angular velocities \dot{\phi}_{n,0} are not specified. Also, given that this is a non-standard model, it would be very informative for the reader to see some time series of the phases themselves to build a better mental image of the system. Alternatively, the authors could use a more well-known oscillator model, such as coupled Kuramoto oscillators. For example, one particularly attractive domain of application would be metastable chimera states [4]”**.

We have revised the detailed parameter settings in the Method section and Result section (Line 163-167):

*In this paper, the initial phases are set randomly, and the phase-locking strength a_mn is uniformly set as 0.1. The initial phase increment determines the initial position of the oscillator in the angular speed time series, which would be described specifically in each simulation scenario.*

In Fig 4 we have shown the angular speed time series, which should be a clear informative representation of the oscillator evolution.

12. Reviewer #2 wrote **“The quality of the figures could be substantially improved. They are very clearly the default Matlab settings, which are not of publication quality. There are also a few particular issues in some figures, for example: in Figs. 3 and 4, it is very confusing that the X-axis for two subplots is time, but in the third plot it is entry index; Fig. 7 has a continuous colour bar but discrete values; Fig. 9 has tiny fonts and no subfigure labelling. Given that PLoS ONE does not apply any copyediting to the paper, the authors should spend significant effort on it (for example, positioning of figures 5,6,8, font sizes in figures 9,10).”**

Thank you very much for your comments on the figures. In Fig 7 we have revised the color bar. In Fig 9 we have added the subfigure label and increased the font size. In Fig 12 we have increased the font size.

13. Other minor revision:

We have made sure that the authors received no specific funding for this work and removed the irrelevant part in Acknowledgement.

We have revised the ethical statement in the ethical section and the caption of Fig 2 as: *The individual in this manuscript has given written informed consent (as outlined in PLOS consent form) to publish these case details.*

The above revisions have been highlighted in document named as 'Revised Manuscript with Track Changes'. We would like to thank you for your suggestions and comments for this manuscript and our research again.

Sincerely yours,

Xinzhe Li

on behalf of all the authors
